# Supplementary material for: Altered microRNA Transcriptome in Cultured Human Liver Cells upon Infection with Ebola Virus
Source: Int J Mol Sci. 2021 Apr 6;22(7):3792. doi: 10.3390/ijms22073792 (PMC8038836; doi:10.3390/ijms22073792)
Supplement: Supplementary file 1 [file ijms-22-03792-s001.zip › Supplementary_File/D_ KEGG_Analysis_Results/16-30nt_pathway_Mayinga-24h-Huh7_vs_Control-24h-Huh7_down.mature_mirna_targets/hsa_EnrichmentScoreDotPlot.pdf]

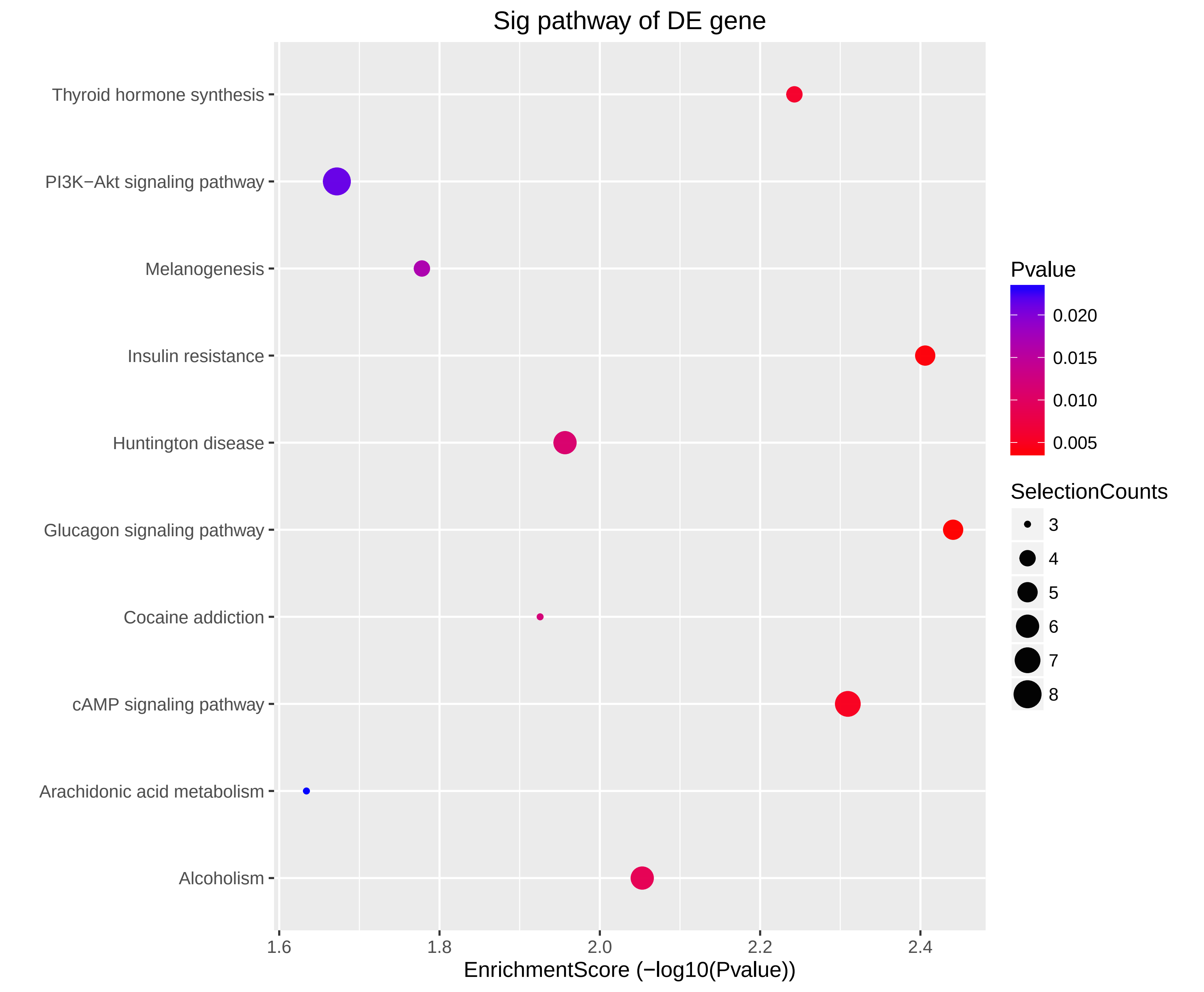

Sig pathway of DE gene
Thyroid hormone synthesis
PI3K−Akt signaling pathway
Melanogenesis
Insulin resistance
Huntington disease
Glucagon signaling pathway
Cocaine addiction
cAMP signaling pathway
Arachidonic acid metabolism
Alcoholism
1.6
1.8
2.0
2.2
2.4
EnrichmentScore (−log10(Pvalue))
Pvalue
0.020
0.015
0.010
0.005
SelectionCounts
3
4
5
6
7
8
